# Mapping Barriers and Interventions to Diabetes Self-Management in Latino Youth: A Scoping Review

**DOI:** 10.3390/children12070882

**Published:** 2025-07-03

**Authors:** Milena de Lucca, Megan Visser, Tatiane Geralda André, Sisi Namoc Leturia, Lucila Castanheira Nascimento, Rebecca Ortiz La Banca Barber

**Affiliations:** 1 College of Nursing, University of São Paulo at Ribeirão Preto, São Paulo 05508-220, Brazil; milenalucca@usp.br (M.d.L.); tatianeandre@usp.br (T.G.A.); lucila@eerp.usp.br (L.C.N.); 2 Institute for Nursing and Interprofessional Research, Children’s Hospital Los Angeles, Los Angeles, CA 90027, USA; mvisser@chla.usc.edu; 3 Center for Endocrinology, Diabetes and Metabolism, Children’s Hospital Los Angeles, Los Angeles, CA 90027, USA; snamoc@chla.usc.edu

**Keywords:** type 1 diabetes mellitus, type 2 diabetes mellitus, pediatric diabetes, diabetes self-management education and support, Latino health, children, adolescent

## Abstract

**Background/Objectives:** Effective diabetes self-management is critical for glycemic management and well-being, yet Latino youth face unique cultural and socioeconomic barriers that are insufficiently explored in the literature. This review mapped existing evidence on diabetes self-management for Latino youth. **Methods:** Searches were conducted in PubMed, CINAHL, SCOPUS, Web of Science, LILACS, ERIC, and The Cochrane Library, using the gray literature and reference lists, in September 2024, following JBI guidelines. The included studies were qualitative, quantitative, and mixed-methods studies and reviews on diabetes self-management for Latinos aged 0–30 with type 1 or 2 diabetes. Studies including participants over 30 or with gestational diabetes were excluded. Two reviewers independently extracted data using a standardized table and analyzed findings using the Association of Diabetes Care & Education Specialists framework (ADCES7) for self-care behaviors: healthy eating, being active, monitoring, taking medication, problem-solving, reducing risks, and healthy coping. **Results:** Forty-five studies (forty from the United States) were included from 860 citations. The findings highlighted challenges in adopting diabetes-friendly diets, including cultural preferences, food insecurity, and limited resources. Physical activity improved glycemic control but was hindered by family and school obligations. Continuous glucose monitoring (CGM) enhanced outcomes, though economic barriers limited access. Family-centered education improved medication adherence, while family support strengthened problem-solving. CGMs and insulin pumps reduced complications, and culturally adapted psychological support enhanced emotional well-being and glycemic management. **Conclusions:** This review underscores persistent disparities in diabetes self-management among Latino youth. While the study designs and settings were heterogeneous, the findings highlight the need for culturally tailored, family-centered interventions that address structural barriers and psychosocial needs to improve care.

## 1. Introduction

Diabetes is a chronic metabolic condition affecting an estimated 537 million people worldwide, constituting a huge public health burden [1]. Based on demographic and incidence trends, it is projected that the number of children and youth with diabetes will increase by at least 12% by 2060. Due to population changes, it is also projected that the number of type 1 (T1D) and type 2 diabetes (T2D) cases will increase among Hispanic/Latino youth as well as among other racial/ethnic minorities [2]. Compared to White youth, Hispanic/Latino youth demonstrate worse HbA1c trajectories over time among youth with T1D or T2D [3]. This group is also disproportionately affected by social determinants of health, including poverty, limited access to culturally and linguistically appropriate care, and structural barriers in the health system, which can exacerbate poor diabetes outcomes [3,4].

Living with diabetes requires daily glucose monitoring, decision-making and frequent complex tasks to manage the illness effectively throughout one’s life. Diabetes self-management is the term used to describe the activities in which youth and their parents share responsibilities and make decisions to achieve management of the illness and overall well-being [5]. Broader than medication adherence, diabetes self-management activities include healthy eating, being active, glucose monitoring, problem-solving, and risk assessment. Interventions, such as diabetes self-management education (DSME), have been shown to improve several aspects of living with diabetes, such as overall glycemic management, better use of technology, improved quality of life and mental health outcomes [5,6]. Research has identified challenges related to the implementation of DSME, including a lack of infrastructure, family-level factors, and the need for tailoring intervention for specific populations [5,7,8]. Latino families, in particular, may experience unique barriers related to language, cultural health beliefs, immigration-related stress, and inconsistent access to care—all of which can influence the success of self-management programs and youth engagement in diabetes care [3,4].

Systematic reviews have been conducted that focused on youth diabetes self-management related to the following: problem-solving [9,10], digital interventions [11,12,13], financial incentives [14], psychological or psychiatric factors [15,16], skill development [17], and the views of children from underserved communities [18]. There have also been reviews conducted on self-management behaviors and interventions to support US Latinos with T1D and T2D [19,20,21,22,23]; however, little attention has been paid to diabetes self-management among Latino youth specifically.

To our knowledge, no prior review has comprehensively mapped the existing literature on diabetes self-management among Latino children and youth, despite this population’s persistent disparities in clinical outcomes. Given the projected demographic changes and continued underrepresentation of Latino youth in diabetes research, this gap limits the ability of clinicians, educators, and policymakers to design interventions that are culturally responsive and developmentally appropriate. A clearer understanding of the available evidence is essential to support more equitable care, inform family-centered management strategies, and identify priorities for future research. Therefore, the objective of this scoping review is to map the evidence on diabetes self-management for Latino children, adolescents and young adults diagnosed with T1D or T2D, aged 0 to 30 years.

## 2. Materials and Methods

A scoping review was conducted to map the literature on diabetes self-management for Latino children and youth with diabetes following the JBI guidelines [24]: (I) development of the research question; (II) definition of inclusion criteria; (III) development of search strategies; (IV) screening and selection of studies; (V) data extraction; (VI) analysis of evidence; and (VII) presentation of results. The Preferred Reporting Items for Systematic Reviews and Meta-Analyses Extension for Scoping Reviews (PRISMA-ScR) [25] was used to guide this review and the subsequent report. This review was not previously registered.

### 2.1. Development of the Research Question

The “PCC” mnemonic (population, concept, and context) guided the review question. P, for population, includes children, adolescents, and young adults with diabetes who self-identify as Latino/Hispanic. For the purposes of this review, we used the term “Latino” to represent all individuals who live or have lived in, or have emigrated from Latin America and/or hold a strong cultural connection to this region; C, for concept, represents diabetes self-management, and C, for context, was not applied to the search. The formulated research question was as follows: What is the existing evidence regarding diabetes self-management in Latino youth?

### 2.2. Eligibility Criteria

Eligible study designs included original qualitative, quantitative, and mixed-methods studies (e.g., randomized controlled trials, observational studies, case studies, and ethnographies), as well as theoretical papers and reviews on diabetes self-management for Latino youth. For diabetes self-management, this review used the Association of Diabetes Care & Education Specialists framework (ADCES7) for self-care behaviors [26]: (1) healthy eating: understanding and implementing a balanced diet that supports glycemic management; (2) being active: engaging in regular physical activity to improve overall health and manage diabetes; (3) monitoring: keeping track of glycemic levels and understanding how they relate to food, activity, and medication; (4) taking medication: focusing on adherence to prescribed medications and understanding their role in diabetes management; (5) problem solving: developing skills to address challenges and make informed decisions regarding diabetes care; (6) reducing risks: identifying and mitigating potential health risks associated with diabetes; and (7) healthy coping: utilizing strategies to manage the emotional and psychological aspects of living with diabetes.

The population of interest included children, adolescents and young adults diagnosed with T1D or T2D aged 0 to 30 years who identify as Latino. Results of studies discussing other populations comprising specific Latinos/Hispanics were included, as well as studies where more than 50% of the sample comprised Latino/Hispanic individuals. Studies in English, Portuguese, and Spanish were included due to the fluency of the authors in these languages. There was no restriction on the publication year of the included studies. Studies including participants older than 30 years or adults in the same sample, as well as those including gestational diabetes in the sample, were excluded.

### 2.3. Search Strategies

To develop the search strategy, descriptors from DeCS-MeSH, Cinahl titles, and ERIC Thesaurus were consulted. The search strategy combined descriptors and keywords employing the Boolean operators AND, OR, and NOT. Searches were conducted across the US National Library of Medicine (PubMed), Cumulative Index to Nursing and Allied Health Literature (CINAHL), Literatura Latino-Americana de Informação Bibliográfica (LILACS), SCOPUS, Web of Science, Education Resources Information Center (ERIC), and The Cochrane Library. The strategy was tailored to meet the specific requirements of each database, and an example is presented in Appendix A. The search was conducted in September 2024.

The gray literature was sought through Google Scholar publications. Additionally, the reference lists of included studies were manually reviewed to identify other potential studies.

### 2.4. Screening and Selection Process

Identified citations were imported into Covidence^®^ [27] for the screening and selection process. Duplicate records were automatically removed. Title screening and abstract screening were then conducted independently by two reviewers, based on the pre-defined inclusion and exclusion criteria. Any discrepancies between reviewers were discussed and resolved by consensus.

Following this initial screening, full texts of potentially relevant studies were retrieved and assessed independently by the same two reviewers. Conflicts during the full-text review phase were again resolved through discussion and consensus meetings. If consensus could not be reached, a third reviewer was available to make the final decision.

### 2.5. Data Extraction and Analysis of Evidence

Two reviewers independently extracted data from the included studies using a standardized extraction table created within Covidence^®^. Information mapping took place based on the JBI instrument to characterize the studies [28]. The extracted information included the following: the author(s), year of publication, state and country where the study was conducted, study aims, study design and characteristics, specific ADCES7 Self-Care Behaviors addressed, total number of participants, number and percentage of Latino participants, age range, type of diabetes, main findings, and reported limitations. A third reviewer reviewed the extracted data for accuracy and consistency.

Extracted data were grouped and analyzed following the ADCES7 framework. This thematic organization facilitated the identification of patterns, strengths, and gaps across studies. Findings were synthesized and presented through summary tables, figures and narrative descriptions to provide a comprehensive overview of the evidence.

## 3. Results

**Figure 1:**
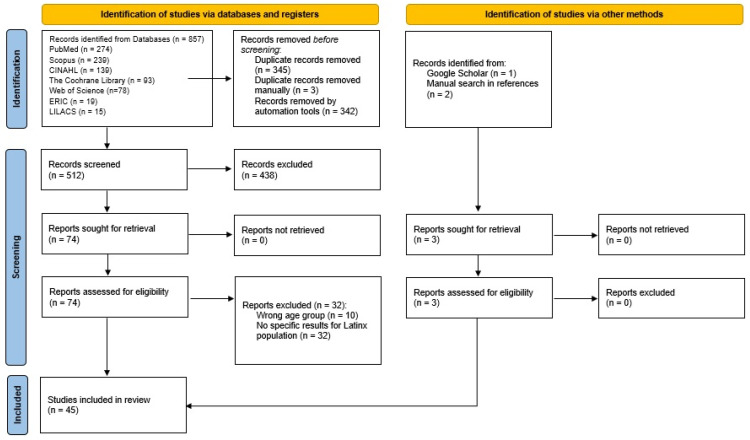
PRISMA flowchart of the screening process (source: [29]).

The original search identified 857 potentially relevant citations, of which 345 were removed because they were duplicates. In total, 512 went through title and abstract screening, and 438 studies were excluded for not meeting the inclusion criteria. Finally, from the 74 full-text articles screened, 45 comprised the final sample of the scoping review: 42 studies were selected through database searches, two studies were retrieved from the reference list, and one article was obtained from Google Scholar. Figure 1 displays the PRISMA flow diagram [29].

### 3.1. Included Studies Characteristics

**Figure 2:**
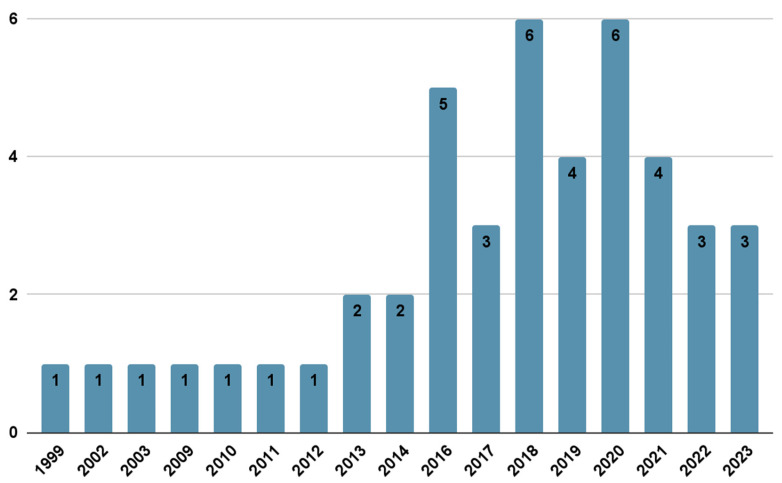
Articles per publication year.

The characteristics (Table 1) of the 45 studies include the fact that a majority were conducted in the United States (n = 40; 88.9%), including states with large Latino populations such as California, Texas, and Florida. Years of publication span from 1999 to 2023 (Figure 2). In terms of study design, most of the included studies are cross-sectional (n = 25; 55.6%), followed by longitudinal (n = 8; 17.8%) and experimental (n = 7; 15.6%) designs. A smaller proportion of studies include qualitative studies (n = 5; 11.1%) and reviews (n = 2; 4.4%). The age range of participants predominantly focuses on adolescents (with a mean age between 12 and 15 years).

### 3.2. ADCES7 Key Areas

Findings include a wide range of factors influencing diabetes self-management among Latino youth, such as disparities in glycemic management, low technology adoption, psychosocial challenges, family dynamics and the impact of culturally tailored interventions. Themes highlight barriers to and facilitators of effective diabetes self-management, with evidence pointing to persistent inequities in access to care and diabetes technology, and potential benefits of family involvement, peer support, and culturally responsive education. It is important to note that some studies were categorized under more than one of the ADCES7 key areas, as they addressed multiple self-care behaviors.

#### 3.2.1. Healthy Eating

Factors influencing the dietary behaviors of Latino youth with T1D and T2D were discussed in seven studies. Findings indicate that Latino youth generally have healthier dietary habits compared to non-Hispanic youth in a cross-sectional study investigating the correlates of dietary intake in youth with T1D and T2D; a sample of 324 Latino adolescents with a mean age of 15 years showed higher fiber and calcium intake and lower soda consumption [37]. Puerto Rican children with T1D reported maintaining healthy eating behaviors with balanced meals within the recommended dietary guidelines [64]. Additionally, eating more meals at home was associated with stronger parent-child relationships [55].

Challenges in maintaining healthy eating habits were also reported. Latino youth experience higher rates of obesity and overweight, which negatively impacts diabetes management [44]. These weight issues are exacerbated by acculturation—the process by which individuals from immigrant populations adopt the cultural norms of their new country—and food insecurity, which refers to the lack of consistent access to enough food for an active and healthy life [44]. These factors contribute to unhealthy dietary patterns, such as high consumption of sugary beverages and low intake of fruits and vegetables [44]. Traditional Hispanic foods, often rich in carbohydrates, present difficulties in balancing cultural food preferences with diabetes management. Moreover, adolescents noted that they struggled to make healthy choices when in social environments like school, where unhealthy food options were common [3,61].

Family involvement plays a key role in shaping dietary habits. Studies indicated that Latino youth who shared diabetes care responsibilities with their parents had better eating habits than those managing care independently [55]. However, language barriers, particularly in families with limited English proficiency, can negatively impact adherence to dietary and medical recommendations. In a study assessing access to and interest in smartphone technology for managing T1D in primarily Hispanic adolescents and their parents, 37% of Latino adolescents reported using smartphone applications to assist with carbohydrate counting [63].

#### 3.2.2. Being Active

Physical activity behaviors among Latino youth with T1D and T2D were examined in four studies. Findings indicate that maintaining consistent physical activity presents several challenges for this population, despite its well-established role in glycemic management. In a qualitative study involving Hispanic adolescents with T1D, participants described the ongoing difficulty of balancing exercise with insulin management and food intake [61]. Factors such as puberty, illness, and unpredictable schedules added to the complexity of this task. Adolescents also reported that school obligations and other daily responsibilities often interfered with opportunities for physical activity. Notably, while adolescents emphasized physical activity as a central concern in diabetes self-management, healthcare providers interviewed in the same study discussed it less frequently, suggesting a disconnect between patient experience and provider priorities [61].

In a study of Puerto Rican children with T1D, average physical activity levels were reported to be low, with children engaging in exercise approximately once per day for about 18 min—below recommended guidelines [64]. Poorer metabolic outcomes were associated with both shorter exercise duration and lower frequency; gender differences were also observed, with boys reporting longer periods of physical activity than girls [64].

Engagement in physical activity was also associated with healthier lifestyle habits more broadly. In a cross-sectional study, Latino youth who were more physically active also demonstrated better dietary behaviors, including higher fiber intake and lower soda consumption [37].

An intervention study involving a 19-year-old Mexican-American male with T2D reported increased physical activity and improvements in overall diabetes self-care. After participating in an 8-week culturally tailored, occupation-based program that included activities such as biking, swimming, basketball, and yardwork, his physical activity increased from one to seven hours per week. The intervention effectively integrated exercise into daily routines and was supported by family involvement, resulting in significant improvements in both physical activity engagement and overall diabetes self-care [57].

#### 3.2.3. Monitoring

Fourteen included studies explore blood glucose monitoring practices, glycemic management outcomes, and the use of diabetes technology—particularly CGM systems.

Several studies point to significant disparities in monitoring behaviors and outcomes between Latino youth and White non-Hispanic WNH peers. In a longitudinal cohort study conducted in California, Addala et al. [30] found that Hispanic youth newly diagnosed with T1D and provided with CGM had consistently higher HbA1c levels than their NHW peers. Similarly, Gallegos-Macias et al. [43] and Nicholl et al. [42] reported lower blood glucose monitoring frequency among Hispanic children, although family supervision of diabetes treatment appeared to be stronger, potentially mitigating some negative outcomes.

In resource-constrained contexts, such as diabetes care in Puerto Rico, children with T1D reported engaging in reasonable monitoring behaviors—averaging two blood glucose tests daily—highlighting the role of family resilience in the face of economic adversity [64].

CGM use was notably lower among Hispanic youth across multiple studies [40,41,42,73]. Nonetheless, Hispanic youth who adopted CGM demonstrated improved glycemic outcomes. Ravi et al. [59] found that Hispanic participants who consistently used CGM had lower HbA1c levels (8.5%) compared to non-users (9.3%) and better time-in-range metrics. Similarly, Baboun et al. [34] reported better overall glycemic management among technology users, with an HbA1c of 8.4% versus 9.6% among CGM non-users. Among technology non-users, Hispanic participants still had better glycemic management than Black peers, suggesting additional complexity in how race and technology access interact.

The barriers to CGM adoption in this population appear to be multifactorial. Studies identified socioeconomic status (SES) as a major confounding factor. When SES was accounted for, differences in glycemic management between Hispanic and White youth often diminished [32,51], indicating that income, insurance type, and healthcare access heavily mediate outcomes in technology use. However, Lipman et al. [71] noted that disparities in both technology use and glycemic management persisted even when insurance type was accounted for, suggesting the influence of structural inequities, cultural factors, and potential provider bias.

Attitudes toward diabetes technology also varied by ethnicity and language. Tsai et al. [67] observed that Latino youth who prefer communication in English expressed more negative views toward CGM and were less likely to use the device compared to Spanish speakers and non-Latino English speakers. This group also reported the highest HbA1c levels (9.69%), indicating a potential link between attitudes toward technology use and glycemic outcomes.

Encouragingly, experimental studies demonstrated that culturally tailored programs could promote better monitoring behaviors. For instance, Bisno et al. [35] showed that Hispanic youth who participated in virtual peer group (VPG) interventions experienced significant HbA1c reductions (−2.76%) and a fourfold increase in CGM usage—from 11% at baseline to 47% by the end of the study. These findings suggest that supportive, community-based approaches may effectively address both psychological and practical barriers to glycemic monitoring.

#### 3.2.4. Taking Medication

The findings of twelve studies reveal suboptimal medication adherence within the Latino youth population, influenced by socioeconomic factors, age, gender, structural barriers, and cultural perceptions surrounding diabetes treatment and technology use.

Evidence points to consistently lower medication adherence rates among Hispanic youth when compared to their WNH peers. In a large cohort study, Adeyemi et al. [31] reported that Hispanic adolescents had a mean medication possession ratio (MPR) of only 42.5%, which is significantly lower than the 50.04% observed among White participants. Adherence also declined with age, and males demonstrated higher MPR than females. Persistence with medication—defined as the duration of time before discontinuation—also declined with increasing age and was lower in Hispanic participants. These trends were echoed in studies examining insulin pump use, where Hispanic youth consistently had lower usage rates compared to WNH peers, even after adjusting for socioeconomic status and healthcare factors [32,33,62]. For example, Agarwal [32] found that only 39% of Hispanic youth used an insulin pump compared to 72% of WNH youth.

Disparities in technology utilization were linked to differences in clinical outcomes. In a retrospective chart review conducted in Pennsylvania, Lipman et al. [71] found that Hispanic youth had 3 times higher odds of not using insulin pumps and 2.7 times higher odds of poor glycemic outcomes compared to WNH youth, even after adjusting for insurance type. These findings reinforce earlier observations by the same author in a 2016 literature review, which reported that Hispanic youth were less likely to receive intensive insulin regimens, contributing to poorer glycemic management relative to WNH peers, though still achieving better outcomes than Black non-Hispanic youth [72].

Youth and caregiver perspectives on medication use highlighted the burden and complexity of insulin management. In a qualitative study, Latino adolescents described insulin administration as a daily struggle, underscoring the emotional and logistical challenges of taking medication self-care behavior [61]. Streisand et al. [64] reported relatively good self-care behaviors among children in Puerto Rico, with girls being more consistent in their insulin administration than boys, suggesting gender-related differences in treatment engagement.

Attitudes toward insulin and related technologies emerged as another influential factor. As noted earlier in the study by Tsai et al. [67], which highlighted negative perceptions of CGM among English-speaking Latino youth, similar attitudes were observed toward insulin pumps. These adolescents also showed greater reluctance to adopt insulin delivery technologies, reinforcing the role of language and cultural context in shaping engagement with diabetes management tools. Despite lower utilization, interest in diabetes technology was evident in other studies. George et al. [63] found that both Hispanic adolescents and their parents expressed moderate to high interest in smartphone apps for insulin dose calculation, with parents showing even greater enthusiasm than their children.

In a cohort retrospective study by Vajravelu et al. [69], clinical escalation of therapy—transitioning from non-insulin to insulin-based regimens—was more frequently observed among Hispanic youth with T2D. The authors found that this group was nearly twice as likely to require treatment intensification compared to their WNH peers. Notably, this pattern was especially pronounced among older adolescents and those who were more adherent to metformin, suggesting that even with initial medication adherence, glycemic management in Hispanic youth may still necessitate earlier or more aggressive insulin-based intervention.

#### 3.2.5. Problem Solving

Problem-solving behavior is addressed in ten studies which examine the capacity of Latino children, adolescents, and young adults with diabetes and their families to develop and apply skills necessary to navigate challenges in diabetes care. The findings of our review suggest integrating self-care into daily life, managing diabetes in social and school settings, responding to barriers such as limited access to culturally competent care, and building confidence in self-management.

Shared responsibility between caregivers and youth emerged as a key factor associated with better self-management. Bolter et al. [36] found that, although Latina mothers reported more shared and less adolescent-only diabetes care responsibility than WNH mothers, shared responsibility—regardless of who initiated it—was associated with improved self-management behaviors, particularly among Latina families. Similarly, Gandhi (2016) [44] found that this pattern may reflect the influence of *familismo*, a cultural value emphasizing family interdependence, which was linked to greater caregiver involvement in diabetes-related tasks, but also to delayed autonomy in youth decision-making regarding diabetes management.

Adolescents often face psychosocial barriers to problem solving, particularly in navigating their diabetes among peers and in school environments. Joiner et al. [3] reported that both adolescents and their parents initially lacked critical knowledge and skills needed for diabetes management following diagnosis. Adolescents described social stigma and logistical challenges as obstacles to effective self-care, including skipping glucose checks to avoid standing out at school or missing class. Parents, while supportive, sometimes struggled to balance reminders with their children’s desire for independence, highlighting the complex emotional dynamics in diabetes problem solving.

Several studies emphasized the positive impact of culturally tailored interventions in enhancing problem-solving skills and glycemic outcomes. The Spanish Language Diabetes Clinic (SLDC) demonstrated notable improvements in glycemic management compared to an English-language clinic, suggesting that linguistically and culturally concordant care can facilitate better understanding and management of diabetes [54]. Similarly, a culturally sensitive Shared Medical Appointment (SMA) model led to reduced HbA1c levels and increased insulin pump use, especially among younger children, with families expressing high satisfaction with the culturally aligned care model [56].

Occupation-based interventions like the REAL Diabetes program also showed promising outcomes. These interventions focused on building problem-solving habits and integrating diabetes care into participants’ daily routines. Piven [57] documented improvements in adolescents’ self-efficacy, blood glucose monitoring, and physical activity following a tailored intervention. Pyatak et al. [58] expanded this model in a randomized controlled trial with significant improvements in HbA1c and self-care habits among low-income, predominantly Latino young adults with T1D and T2D. Participants in the intervention group increased their blood glucose frequency and reported better quality of life.

Furthermore, innovative models such as the CoYoT1 telehealth intervention for youth with T1D demonstrate potential to benefit the Latino youth population. While glycemic outcomes did not significantly improve, study participants exhibited increased clinic attendance, had reduced diabetes distress, and had greater satisfaction with the flexibility of telehealth appointments [60]. This suggests that problem solving may extend beyond direct clinical outcomes to include logistical and emotional barriers to consistent diabetes care.

Finally, the culturally adapted Familias Apoyadas intervention highlighted the importance of peer mentorship and culturally relevant education. Latino parents of young children with T1D benefited from bilingual materials, culturally appropriate dietary adaptations, and social support from trained mentors. This approach addressed common barriers such as language differences, difficulty navigating school systems, and lack of culturally competent providers [65].

#### 3.2.6. Reducing Risks

Nine studies examined diabetes complications and social determinants of health, including SES, healthcare access, and family support, all of which play critical roles in either mitigating or exacerbating health risks in this population.

Many highlighted the strong connection between socioeconomic disadvantage and poorer diabetes outcomes. Gallegos-Macias et al. [42] found that while Hispanic families had significantly lower income and educational attainment, low socioeconomic status was most strongly associated with elevated HbA1c as opposed to ethnicity. Gandhi [44] similarly reported that Latino youth often face barriers such as lack of insurance, limited access to bilingual healthcare providers, and lower health literacy, all of which contribute to suboptimal glycemic management and increased risk of complications. Chang et al. [40] emphasized that poor glycemic management in the first year following diagnosis is a strong predictor of long-term outcomes, noting that youth who presented with an initial HbA1c >8.5% had a two-fold higher risk of presenting poorly managed diabetes five years later.

Family support emerged as a protective factor, particularly when responsibility for diabetes management was shared and guided by parental involvement. Hsin et al. [47] found that better adherence was associated with more family involvement, higher parental education, and lower levels of youth independence in diabetes tasks—suggesting that premature transfer of responsibility may increase the risk of poor outcomes. This protective role of family was further supported by Taylor et al. [66], who found that youth satisfaction with healthcare providers, especially in terms of communication and relationship quality, was significantly linked to better adherence to treatment regimens, an essential factor in preventing complications. Notably, this effect was stronger for girls, and there were no significant differences in provider satisfaction across ethnic groups.

Clinical interventions tailored to Latino youth demonstrated meaningful reductions in risk-related outcomes. A culturally sensitive SMA model resulted in fewer hospitalizations and emergency department visits and yielded substantial healthcare cost savings [45]. Similarly, interventions that incorporated bilingual education and culturally relevant care strategies were associated with improved metabolic management and greater engagement in self-management behaviors [44].

Glycemic variability and its immediate consequences were also examined as risk factors for academic performance. Knight et al. [48] found that both hyperglycemia and hypoglycemia negatively impacted reading, writing, and math fluency among youth with T1D, suggesting that high glucose variability not only increases the risk of long-term complications but may also hinder cognitive and educational development.

Importantly, Reitblat et al. [61] noted that language barriers were not perceived by adolescents as a major obstacle to care, even when translating for family members during clinic visits. However, clinicians often viewed these barriers as more significant, pointing to a potential disconnect between provider perceptions and patient experiences—an important consideration in risk reduction efforts.

Finally, while Hispanic youth present higher HbA1c levels than White youth, their outcomes were generally better than those of Black youth, placing them at an intermediate level of risk. Alan et al. [70] reported that although 46.9% of Hispanic youth had poor glycemic management, their rates of DKA hospitalization were comparable to those of their White peers and lower than those of Black youth.

#### 3.2.7. Healthy Coping

Healthy coping behavior was addressed in fourteen studies which explore how Latino youth and their families manage the emotional, psychological, and social demands of living with a chronic condition.

Psychosocial concerns are widespread among Latino youth with diabetes, with substantial links to clinical outcomes. Brodar et al. [38] found that over 75% of adolescents screened in a pediatric diabetes clinic endorsed at least one psychosocial concern, such as low motivation, non-adherence to insulin regimen, depressive symptoms, anxiety, or diabetes-related stress. These concerns were significantly associated with higher HbA1c levels, family conflict, disordered eating, and emotional distress.

Stress, stigma, and depressive symptoms were also prominent themes across other studies. Butler et al. [39] found that diabetes-specific stress was more prevalent and more strongly associated with poor glycemic management among Hispanic emerging adults compared to their WNH peers. Similarly, Ramos [41] reported that over two-thirds of Latino adolescents experienced diabetes-related stigma, which was significantly linked to higher rates of depressive symptoms, particularly when stigma came from peers or was internalized. These emotional burdens were compounded by cultural and structural stressors, as described by Gandhi [44], who noted that Latino youth often experience family conflict, underutilization of mental health services, and limited access to culturally competent care.

Family relationships played a complex role in emotional coping [46]. While *familismo* often facilitated self-care and emotional regulation [49,50], mismatches in perceived stress between adolescents and their parents, as observed by Mello [51], were linked to worse diabetes outcomes. Adolescents whose mothers underestimated their stress or coping competence had higher HbA1c, suggesting that caregiver attunement to emotional needs may be critical for effective disease management.

Despite the challenges, several protective coping mechanisms emerged in this review. Many Latino families emphasized spirituality as a central source of strength. Joiner et al. [3] found that both adolescents and their parents relied on religious beliefs and community support to manage diabetes-related stress, with adolescents often expressing that their faith and spiritual communities helped them cope. Cultural values around family well-being also motivated youth to adhere to treatment despite stigma or embarrassment.

Open communication and emotional self-efficacy were further identified as key elements of healthy coping. Tucker et al. [68] found that adolescents who disclosed their feelings and diabetes-related challenges to their parents had better glycemic management, improved adherence, and fewer depressive symptoms. In contrast, secrecy—particularly from fathers—was linked to worse outcomes. Pagán-Torres [53] showed that higher emotional self-efficacy, as measured by a validated scale for Latino youth, was associated with greater life satisfaction, improved diabetes self-care, and stronger family support.

Interventions that addressed emotional and cultural needs demonstrated positive effects on coping and diabetes outcomes. The REAL Diabetes program, discussed in earlier categories, also contributed to healthy coping by integrating mental health and emotional regulation into self-management training [60]. Similarly, Sullivan-Bolyai [65] adapted the STEP parent-mentor intervention into Familias Apoyadas, offering culturally relevant peer support to Latino families of children with T1D. The program addressed challenges such as language barriers, emotional burden, and cultural food practices, offering tools for coping that were rooted in cultural strengths.

**Figure 3:**
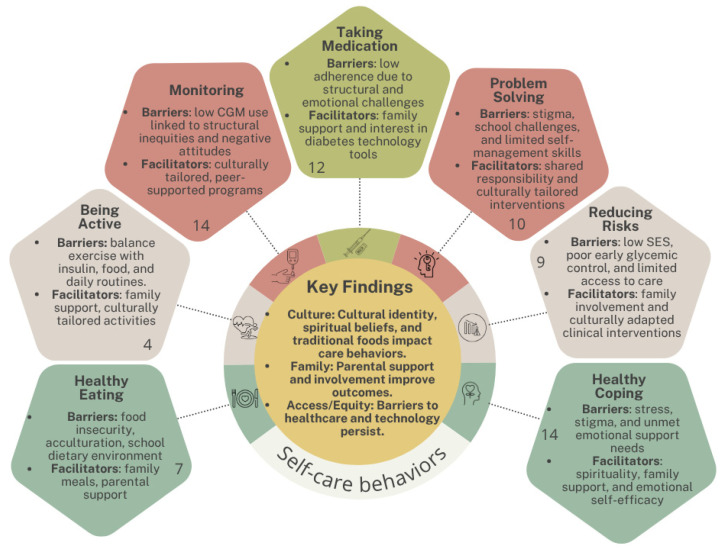
Summary of key findings across ADCES7 self-care behaviors among Latino youth with diabetes.

Figure 3 summarizes the main findings of the scoping review related to the seven self-care behaviors defined by the ADCES7 framework: healthy eating, being active, monitoring, taking medication, problem solving, reducing risks, and healthy coping. Each colored hexagon represents one of these behaviors and includes a brief description of the most commonly reported barriers and facilitators. The number inside each hexagon indicates how many studies from the review addressed that specific self-care domain. At the center of the figure, key cross-cutting findings are highlighted: the influence of culture, the critical role of family, and persistent challenges related to access and equity in diabetes care and technology use.

## 4. Discussion

This scoping review mapped the existing evidence on diabetes self-management among Latino youth with T1D or T2D, revealing both the strengths and substantial barriers across the seven ADCES self-care behaviors. The findings converged on a core implication: the critical need for interventions that are culturally responsive, family-centered, and attuned to the psychosocial and structural realities faced by Latino youth. These three pillars—culture, family, and psychosocial context—intersect with persistent inequities in access to diabetes care and technology, shaping both challenges and opportunities for improving health outcomes. The interpretation of our findings supports the broader recognition that successful diabetes self-management among Latino youth requires multilevel strategies that go beyond individual behaviors to address the social determinants of health.

Culturally responsive interventions showed promise in improving diabetes self-management among Latino youth in areas such as monitoring, problem solving, and risk reduction [45,54,56]. Cultural factors, such as values around family well-being or spirituality, can play an important role in healthy coping [3], while acculturation of immigrant Latino youth with T1D or T2D may exacerbate challenges with diabetes self-management, such as healthy eating [44]. Interventions that integrated cultural understanding—such as those that acknowledged traditional foods, language preferences, and family norms—tended to facilitate greater adherence and improved metabolic outcomes [3,44,74]. For example, programs like REAL Diabetes [60] and Familias Apoyadas [65] successfully addressed language barriers, emotional burden, and culturally specific food practices by offering peer mentorship and psychoeducation grounded in cultural strengths. Rather than treating cultural tailoring as an add-on, these programs demonstrate that it must be a foundational component of intervention design. Cultural tailoring should extend beyond linguistic translation to encompass broader sociocultural elements, including health beliefs, literacy levels, socioeconomic realities, and values [75]. As Fortmann et al. [75] emphasize, interventions that incorporated these deeper cultural dimensions were more successful in improving behavioral and clinical outcomes, whereas those relying only on surface-level adaptations often failed to address psychosocial needs effectively.

The role of the family was another consistent theme across studies, playing important and complicated roles in the diabetes self-care of Latino youth. Our evidence showed that youth with more family involvement in diabetes care presented better eating habits [55], reasonable monitoring behaviors [42,43,64], improved problem-solving behaviors [36,44], better management of health risks [47,66], and some aspects of healthy coping [49,50]. However, this involvement could be both beneficial and complicated, especially in the presence of misaligned stress perceptions between parents and adolescents [51]. The cultural value of family cohesion (*familismo*, or familism) often facilitated support but also occasionally conflicted with adolescents’ growing need for autonomy. Based on our review, family-centered interventions should address parental attunement to youth stress levels, parent-youth communication, relationships with clinicians, difficulties navigating school systems, and the need for youth autonomy in diabetes-related decision-making as they transition into young adulthood [44,51,65,66,68]. These findings reinforce the need for family-centered approaches that are developmentally sensitive and responsive to cultural values.

Psychosocial dimensions of diabetes self-management for Latino youth include issues of emotional self-efficacy, social struggles, and diabetes-related stigma, as well as navigating peer relationships or school environments. These concerns are associated with worse health outcomes, greater emotional distress, and increased family conflict [38,41]. Our sample suggested that Latino youth experience psychosocial concerns, such as emotional struggles related to daily medication use [61] and the use of insulin pumps [67], or diabetes-related stigma with peers [41]. It also identified social obstacles to self-management of diabetes among Latino youth, especially at school or with peers [3]. Youth who demonstrated greater emotional self-efficacy and who received emotional support from family or peers tended to report better quality of life and more consistent engagement in care [74]. Virtual peer groups emerged as a potentially effective intervention for supporting Latino youth self-management of diabetes, such as improving monitoring behaviors [35]. Other interventions, such as the REAL Diabetes program [60] and Familias Apoyadas [65] also offered tools to address psychosocial challenges faced by youth with diabetes. However, consistent with Fortmann et al. [75], psychosocial outcomes may be more resistant to change than behavioral or clinical indicators, suggesting that effective interventions may require more intensive or targeted mental health components to address emotional distress and diabetes-related stigma.

Persistent inequities in access to care and technology make it challenging for Latino youth to use diabetes self-care practices. In our review, language barriers [63], lack of access to smartphone applications [63], and food insecurity [44] influenced dietary and monitoring behaviors. Socioeconomic barriers influenced self-monitoring practices and risks of diabetes complications [40,42,44] among Latino youth. Disparities in the availability and use of diabetes technology—particularly CGMs—were evident, with Latino youth less likely than their WNH peers to receive such tools, even when accounting for insurance status. Our review suggested that increased access to diabetes technology for Latino youth holds promise for improving diabetes self-management. Even with lower usage of diabetes technology than their WNH peers, Latino youth who consistently used CGM showed improvements in glycemic management compared to non-users [59]. Confronting potential clinician bias and structural factors may be another important step to reduce barriers in monitoring as well as in taking medications. Addressing these disparities requires not only expanded access to diabetes technology, but also structural changes—such as clinician training in cultural humility, investment in community-based care infrastructure, and the integration of human interaction through *promotores* or community health workers into technology-based interventions, which can enhance engagement and bridge access gaps for low-income Latino populations with limited digital literacy [75].

Although all seven self-care behaviors were represented to some extent in the literature, physical activity stood out as the least discussed, despite its known benefits for glucose management. While the importance of physical activity in diabetes management is well established, multiple studies underscored that youth—particularly Latina girls—face a range of individual, familial, and cultural barriers to engaging in regular physical activity. This represents a significant gap in the literature. One relevant example outside the reviewed youth studies is the En Balance program for Spanish-speaking Hispanic adults with T2D, which provided culturally and linguistically tailored diabetes education on nutrition and exercise [73]. Participants in this intervention demonstrated improved A1C levels and increased moderate to vigorous physical activity after three months. These findings suggest that when physical activity is framed within culturally meaningful contexts and made accessible through family-supported routines, it is more likely to be adopted and sustained. Adaptation of such programs for youths should be a focus of future research.

Taken together, the findings from this review suggest that addressing diabetes self-management in Latino youth requires multidimensional, equity-driven strategies that attend to culture, family, mental health, and systemic access. Programs that are effective tend to be those that view youth within their social and cultural environments rather than in isolation. These results align with previous studies suggesting that structural and psychosocial determinants significantly influence self-care behaviors and outcomes among marginalized populations. Future research should explore the scalability of culturally and linguistically adapted interventions, particularly those that incorporate family engagement, peer support, and mental health components. As Fortmann et al. [75] highlight, interventions must also attend to the diversity within the Latino population—including national origin, immigration status, and health beliefs—to ensure acceptability and sustainability. There is also a need for more research on underrepresented aspects of self-care—especially physical activity—and on how intersectional factors such as gender, immigration status, and socioeconomic inequality intersect with diabetes care. Efforts that combine clinical, emotional, and structural components in a culturally grounded way may be best positioned to improve long-term outcomes for Latino youth living with diabetes.

## 5. Conclusions

This scoping review offers a comprehensive synthesis of the literature on diabetes self-management among Latino youth, highlighting both challenges and strengths across all seven self-care behaviors. A major strength of this review is its systematic and structured approach to mapping the breadth of available evidence, which allows for the identification of patterns across diverse study populations, settings, and intervention types.

However, as with any scoping review, limitations should be noted. While we employed a comprehensive search strategy across multiple databases, it is possible that relevant studies were missed due to publication bias, incomplete indexing, or language restrictions. Moreover, many of the included studies were cross-sectional and varied in design, limiting the ability to draw comparisons or understand long-term outcomes. The predominance of U.S.-based research may also restrict the generalizability of findings to Latino populations in other contexts. Because the goal of a scoping review is to map rather than critically appraise or synthesize evidence, the findings reflect the scope and descriptive nature of the included studies without assessing their methodological quality or effect sizes.

Despite these limitations, the findings offer important implications for practice. Culturally responsive and family-centered care approaches that integrate emotional and psychosocial support, address structural barriers, and promote equitable access to diabetes technology are essential to improving outcomes among Latino youth with T1D or T2D. Interventions that reflect the lived realities of Latino families—through language access, attention to cultural values, and inclusion of parents and peers—may offer more sustainable and effective models of care.

## Figures and Tables

**Table 1:** Characteristics of included studies.

Reference, Country/State, Objective	Study Design, Latino Participants and Type of Diabetes	Main Results	Limitations
Addala et al. (2023) [30] California, United States of America To determine whether HbA1c decreases differed by ethnicity and insurance status among a cohort of youths newly diagnosed with type 1 diabetes (T1D) and provided with CGM.	Prospective, longitudinal cohort study 29 Latino participants (21.48%), Mean age: 9.7 years (IQR, 6.8–12.7) T1D	Hispanic participants had higher HbA1c levels than non-Hispanic participants at all time points post-diagnosis (6, 9, and 12 months). The increase in HbA1c at 6, 9, and 12 months for Hispanic youths was 0.63% (significant), with a 1.39% HbA1c difference at 12 months. At 12 months, 47% of Hispanic participants achieved an HbA1c < 7.5%, and 47% achieved <7.0%, showing improvement over the historical cohort.	Single-site study design, limiting generalizability; exploratory nature, focusing on ethnicity and insurance status without evaluating racial differences (e.g., absence of non-Hispanic Black participants); small sample size; inability to stratify HbA1c changes by ethnicity or insurance; digital divide impacting CGM data uploads, especially in Hispanic youths.
Adeyemi et al. (2012) [31] TX, United States of America To describe oral antidiabetic medications use and assess trends in medication adherence and persistence among Texas pediatric Medicaid patients.	Retrospective, descriptive study 1869 Latino participants (60%), Median age 14.2 years (SD, 2.3) Type 2 diabetes (T2D)	Medication Adherence: The mean medication possession ratio (MPR) was 44.69% (SD: 27.06%). White participants had the highest adherence (50.04%), followed by Black participants (44.24%) and Hispanic participants (42.50%). Males had higher adherence (47.47%) than females (43.29%). Younger patients (ages 10–12) had the highest adherence (48.82%). Persistence: The mean time to non-persistence (gap of 45 days in medication use) was 108 days (SD: 86 days). Persistence declined with age, with older adolescents (16–18) having shorter persistence.	Lack of generalizability due to the study design; MPR used as a proxy for adherence, which may only reflect medication refills; other covariates not described due to retrospective design; some treatments may have been prescribed for obesity, not T2D.
Agarwal et al. (2020) [32] California, Florida, Texas, Michigan, Pennsylvania, Colorado, United States of America To describe racial-ethnic disparities among young adults (YA) with T1D and identify drivers of glycemic disparity other than socioeconomic status	Cross-sectional multicenter Study 103 Latino participants (34%) Median age 20 years (IQR, 19–20) T1D	The mean HbA1c for Hispanic youth was 9.3 ± 2.2%. Compared to White participants, Hispanic participants had significantly worse socioeconomic indicators, including lower annual household income, lower education, lower social status, and higher neighborhood poverty. In total, 39% of Hispanic participants used an insulin pump, compared to 72% of White participants, and 37% had ever used a CGM, compared to 71% of White participants. After adjusting for socioeconomic status, Hispanic youth showed similar glycemic control to White youth, with no significant differences.	The study was cross-sectional, limiting causal interpretations. The use of English-only materials may have led to the presence of a more educated and acculturated Hispanic population in the sample. Additionally, more Hispanic participants were from pediatric centers, which may have provided better support compared to other settings.
Agarwal et al. (2021) [33] California, Florida, Texas, Michigan, Pennsylvania, Colorado, United States of America (1) To measure the degree of racial-ethnic disparity in insulin pump and CGM use between NH White, NH Black, and Hispanic YA; (2) to examine how multiple factors related to SES contributed to disparities; (3) to determine how patient-reported outcomes such as healthcare factors (care setting, clinic attendance) and diabetes self-management (diabetes numeracy, self-monitoring of blood glucose, and Self-Care Inventory [SCI] score) accounted for disparities.	Cross-sectional multicenter study 103 Latino participants (34%) Median age: 20 years (IQR, 19–20) T1D	Hispanic participants had lower rates of insulin pump (39%) and CGM use (37%) compared to WNH participants (72% and 71%, respectively). After adjusting for SES, healthcare factors, and self-management, the adjusted rates for insulin pump use were 49% and those for CGM use were 58% in the Hispanic group.	The study was cross-sectional, limiting causal conclusions. The use of self-reported data may not fully reflect actual behaviors. The study did not assess factors such as racial discrimination or implicit bias, which may influence disparities in technology use.
Baboun et al. (2023) [34] Florida, United States of America To examine the impact of CGM use and continuous subcutaneous insulin infusion on glycemic control in a racial-ethnic minority cohort of children and adolescents with T1D.	Cross-sectional study 111 Latino participants (79%) Mean age: 12.97 years (SD, 3.25) for technology users and 14.09 years (SD, 3.56) for technology non-users T1D	Technology users had significantly better glycemic control than technology non-users, with a mean HbA1c of 8.40% compared to 9.60% (*p* = 0.0024). Among the technology non-users, Hispanic participants had better glycemic control than Black participants (mean HbA1c of 9.19% vs. 11.26%, *p* = 0.0385). No significant differences in HbA1c were found between Hispanic and Black participants in the technology groups (CGM + MDI, FS + CSII). A total of 27 patients (19%) achieved the ADA’s recommended HbA1c target of less than 7.0%.	The study had an imbalanced representation of ethnic groups, with a disproportionate number of Black and White patients compared to Hispanic patients. There was an absence of data on disordered eating behaviors (leading to insulin misuse for weight management) and depression. Additionally, there was no systematic method to assess the socioeconomic status (SES) of each patient and pair this information with HbA1c levels.
Bisno et al. (2023) [35] California, United States of America To examine the association between attending virtual peer group appointments led by a young adult with T1D and improvements in health-related outcomes within the context of a larger randomized controlled trial of Colorado Young Adults with Type 1 Diabetes (CoYoT1) Clinic, known as CoYoT1 in California.	Longitudinal, experimental study 34 Latino participants (50%) Mean age: 18.15 years (SD, 1.21) T1D	Virtual peer group (VPG) attendees had a significant reduction in HbA1c compared to the standard care group, with a treatment effect of −1.08%. Latinx participants attending VPG sessions showed a larger HbA1c reduction of −2.76% compared to those in standard care. CGM use increased significantly among VPG attendees, from 11% at baseline to 47% by the study’s end, while all other groups showed reduced or stagnant CGM usage.	The study did not follow intent-to-treat principles, analyzing participants by attendance rather than as randomized. Virtual peer group (VPG) sessions led by a youth peer facilitator are not billable, limiting the replicability of this model in other settings. Some patients were excluded, which limits the generalizability of the results. The small sample size also means that a few outliers could significantly influence the mean CES-D score.
Bolter, Main and Wiebe. (2022) [36] Southwestern United States of America To examine whether mother and adolescent reports of responsibility diabetes management tasks varied across ethnic groups. To examine whether shared versus individual (i.e., mother or adolescent) responsibility was linked to better diabetes health outcomes (lower HbA1c, higher self-management behaviors, and fewer depressive symptoms) among Latinx and non-Latinx White families. To test if ethnicity moderated the association between the division of diabetes responsibility and diabetes health outcomes.	Cross-sectional study 56 Latinx mother-adolescent dyads (47.5%) Mean age: 13.24 years (SD, 1.69) T1D	Latina mothers reported more shared and less adolescent responsibility than non-Latinx White mothers, but there were no ethnic differences in adolescent reports of responsibility. Independent of demographic and illness-related characteristics, both mother and adolescent reports of shared responsibility were associated with higher self-management behaviors, while individual responsibility (by either the adolescent or mother alone) was generally associated with lower self-management behaviors. Shared responsibility was associated with higher mother-reported self-management behaviors in Latinx families, but not in non-Latinx White families. Shared and individual responsibility were not associated with HbA1c or depressive symptoms.	The study’s cross-sectional design limits the ability to establish causality between shared responsibility, self-management behaviors, and health outcomes. The reliance on self-reported data from both mothers and adolescents may introduce bias due to discrepancies between their perceptions. Additionally, the study primarily focused on mother-adolescent dyads, with limited data on fathers’ involvement in diabetes management, despite evidence suggesting that fathers may play a unique role in some families.
Bortsov et al. (2011) [37] Ohio, Colorado, Washington, South Carolina, Hawaii, and California, United States of America To explore demographic, socioeconomic, diabetes-related, and behavioral correlates of dietary intake of dairy, fruit, vegetables, sweetened soda, fiber, calcium, and saturated fat in youth with diabetes.	Cross-sectional study 324 Latino participants (11.5% T1D, 20% T2D) Mean age: 15.6 years (range, 10–22) T1D, T2D	Hispanic youth had significantly higher fiber intake compared to White non-Hispanic (WNH) youth. Soda consumption among Hispanic youth was lower than that among WNH youth. Calcium intake was also higher among Hispanic participants than WNH youth.	The cross-sectional design limits causal inferences between correlates and dietary intake. The use of food frequency questionnaires may lead to an underestimation of actual food intake. Additionally, the sample may not be representative, as groups with low socioeconomic status might be underrepresented, which could affect the generalizability of the findings.
Brodar et al. (2021) [38] Florida, United States of America To assess the feasibility and effectiveness of implementing a comprehensive psychosocial screening program in a pediatric diabetes clinic.	Cross-sectional study 136 Latino participants (58.5%) Mean age: 14.82 years (SD, 1.90) T1D	Over 75% of adolescents with T1D screened positive for at least one psychosocial concern. Common issues included low motivation to manage diabetes (52%), insulin non-adherence (36%), mild depressive symptoms (21%), diabetes stress (22%), and anxiety (18%). Psychosocial concerns were significantly associated with clinical outcomes: higher A1c and insulin non-adherence were linked to increased depressive symptoms, anxiety, disordered eating, diabetes stress, family conflict, and blood glucose monitoring stress. Adolescents with worse glycemic control (A1c > 9%) were more likely to have multiple psychosocial concerns, including a higher suicide risk and disordered eating behaviors. Insulin non-adherence, disordered eating, diabetes stress, and family conflict predicted higher A1c. The screening program led to a 25% increase in referrals to the psychology team, suggesting that routine screening helps identify adolescents needing psychological support.	The cross-sectional design limits causal conclusions. The reliance on self-reported data from adolescents may introduce bias and may not fully capture their psychosocial functioning. The absence of parental reports limits the comprehensiveness of the adolescents’ well-being assessment. The exclusion of youth with T2D limits generalizability. The study focused on within-clinic consultations and did not track outcomes for patients referred to external mental health providers. Some measures were modified and unvalidated for this screening program, requiring further validation.
Butler et al. (2017) [39] No specific states, United States of America To examine whether race/ethnicity moderates relationships of (a) diabetes stress and general life stressors with (b) diabetes outcomes of glycemic control and diabetic ketoacidosis (DKA) among emerging adults (aged 18–25 years) with T1D.	Cross-sectional study 357 Latino participants (10%) Mean age: 20.3 years (SD, 2.1) T1D	Hispanic participants had higher HbA1c levels than WNH participants (9.1% vs. 8.4%). Diabetes-specific stress was more strongly associated with higher HbA1c levels among Hispanic participants. In total, 31% of Hispanic participants often or always experienced diabetes-specific stress, compared to 22% of WNH participants. The incidence of DKA was higher in Hispanic participants, with 13% experiencing one or more episodes, compared to WNHs.	It was not possible to examine whether participants with missing HbA1c or DKA data differed from those with complete data. The cross-sectional design prevents us from determining the directionality of the associations. The study did not examine specific general life stressors, and some aspects of diabetes-specific stress were not captured. The use of a one-item measure for general stress is another limitation.
Chang et al. (2020) [40] California, United States of America To (1) analyze glycemic trends and associated risk factors to help identify a high-risk cohort for intensive case management and (2) determine the impact of weight reduction and medication regimen on glycemic outcome.	Retrospective, observational study 183 Latino participants (80%) Mean age: 16.9 years (SD, 2.5) T2D	Weight loss in the first year after diagnosis was associated with improved HbA1c levels, with participants who lost weight showing better glycemic control. Participants on metformin monotherapy had a median weight reduction of 2.6%, while those on insulin-containing regimens gained weight. At 5 years post-diagnosis, the mean HbA1c returned to the initial level, indicating poor long-term glycemic control. The odds of having uncontrolled diabetes (HbA1c > 8%) at 5 years increased if the diagnostic HbA1c was >8.5%, with an odds ratio (OR) of 2.41.	The natural history design lacked uniformity in laboratory tests, and there were missed appointments, which may have affected the power of the results. Some participants had missing clinical data, limiting the ability to draw stronger correlations between HbA1c and variables of interest. The study did not measure beta-cell function or account for changes in medication over time, which might have affected glycemic outcomes.
Crespo-Ramos, Cumba-Avilés and Quiles-Jiménez. (2018) [41] Ponce City, Puerto Rico To explore the prevalence and nature of diabetes-related stigma (DRS) in Latino youth with T1D, focusing on the relationship between stigma and depressive symptoms, as well as the sources of stigma from peers, family, and healthcare professionals	Cross-sectional study 65 Latino participants (100%) Mean age: 15.05 years (SD = 1.68) T1D	Over two-thirds of adolescents (67.69%) reported experiencing at least one form of diabetes-related stigma, with 49.23% experiencing both social stigma (SS) and internalized stigma (IS). Peers were identified as the main source of social stigma. A significant relationship was found between stigma and depressive symptoms, with adolescents experiencing stigma, particularly social stigma, having higher odds of being diagnosed with depression. Depression scores were correlated with the number of stigma experiences. Social stigma included derogatory names like ‘junkie’ or ‘terrorist’ due to insulin use or the use of pumps, while internalized stigma reflected feelings of being different or inferior. Peers were the most frequent source of stigma, followed by family and healthcare professionals. Adolescents from urban areas and larger families were more likely to report stigma experiences.	Sample bias: All participants had depressive symptoms, which may not represent the broader T1D adolescent population. Geographical limitations: The clinic-based setting may have excluded participants from distant regions of Puerto Rico. Contextual influence: Participants may have perceived researchers as therapists, which could have affected how they reported stigma experiences. Instrument limitations: The data collection tool was not specifically designed for stigma assessment, missing factors such as media influence.
Nicholl et al. (2019) [42] No stated specific location from data, United States of America To compare diabetes management trajectories of Hispanic and WNH youth with T1D over the course of late childhood and early adolescence. In addition, the study compared youths’ trajectories on a variety of variables that have been previously shown to impact diabetes management during adolescence (e.g., regimen responsibility, family support, and family conflict).	Longitudinal study 33 Latino participants (13.8%) Mean age: 10.76 years, (SD, 1.06) T1D	At baseline, Hispanic youth had significantly poorer glycemic control, more family conflict, and fewer blood glucose checks on average compared to WNH youth. Similarly to WNH youth, Hispanic youth have increasing independence in regimen tasks and decreasing parent autonomy support during this developmental period. However, while Hispanic youth had worsening diabetes management during early adolescence (as did WNH youth), Hispanic parents reported a more gradual change in youths’ diabetes management over early adolescence.	The focus on Hispanic families in the study treated individuals of different national origins as the same, despite cultural, socioeconomic, and acculturation differences not being assessed. Most Hispanic youth in the sample came from one geographic region, and there are likely important differences among Hispanic subgroups. The small sample size of Hispanic participants in this secondary data analysis limits the study’s confidence and highlights the need for larger, more diverse samples in future longitudinal research.
Gallegos-Macias et al. (2003) [43] New Mexico, United States of America To determine whether there is a disparity in glycemic control between Hispanic and WNH children and adolescents with T1D and to identify factors associated with glycemic control in these populations.	Cross-sectional study 84 Latino participants (45.9%) Mean age: 12.2 years (SD, 4.2) T1D	Hispanic youths with T1D had poorer metabolic control than their WNH counterparts. They exhibited lower compliance with home blood sugar monitoring, but their parents reported greater supervision of their diabetes treatment. Hispanic families had significantly lower incomes, health insurance rates, and educational attainment by both fathers and mothers. Lower family socioeconomic status, but not ethnicity or parental educational attainment, was associated with significantly higher HbA1c, regardless of ethnicity.	The study was conducted in a single clinic, potentially limiting generalizability. It did not examine other ethnic groups beyond Hispanics and White non-Hispanics. The use of self-reported data for some socioeconomic variables may introduce bias.
Gandhi et al. (2016) [44] Authors from Texas, United States of America To discuss the psychosocial aspects of T1D in Latino and Asian-American youth, with a focus on the challenges they face regarding glycemic control, healthcare access, and culturally appropriate interventions.	Literature review T1D	Latino youth with T1D have poorer glycemic control than WNHs, with 65% showing suboptimal control as they age. Socioeconomic status, healthcare access barriers, language issues, and low health literacy affect diabetes management. Family involvement, particularly *familismo*, helps adherence but can reduce youth independence in management. Less acculturated families provide stronger support, but more acculturated youth have worse control. Mental health issues, including depression, are more common and worsened by cultural stressors, yet mental health services are underutilized. Latino youth have higher obesity rates, worsened by unhealthy dietary patterns, contributing to complications. Culturally sensitive interventions, like bilingual education and family-centered approaches, show potential but are scarce.	No direct limitations of the review process are mentioned.
Gold et al. (2021) [45] Colorado, United States of America To evaluate the cost and healthcare utilization (such as emergency department visits and hospitalizations) associated with a culturally sensitive, shared medical appointment model designed for Latino children with T1D. The study also assessed the cost-effectiveness of the program.	Non-randomized controlled trial 57 Latino participants (100%) Mean age 12.6 years (SD, 3.3) T1D	The intervention group had fewer hospitalizations (2% compared to 12% in the control group) and a trend toward fewer ED visits (19% compared to 32% in the control group) 6–12 months after starting the program. The program led to significant cost savings. The per-patient healthcare cost savings were USD 2710 in the first year, with total per-patient savings of USD 2077 when considering both program and healthcare costs. Over five years, the estimated cost savings were USD 14,106 per patient.	Data on medications, test strips, and diabetes-related technology for controls were missing. Technology data were available for program participants, but not controls, raising concerns about the complete ascertainment of technology use. Information on office visits was missing, with difficulty distinguishing diabetes care from shared medical appointments due to multiple diagnosis codes. The study could not identify potential substitutions of shared appointments for regular visits and did not include office visits in the analysis. Time costs for hospitalization, program participation, and travel were not captured, though these are unlikely to outweigh the cost savings from reduced healthcare utilization.
Guerrero-Ramírez and Cumba-Avilés. (2018) [46] San Juan, Puerto Rico To explore the factors associated with suicidal ideation (SI) and diabetes-related quality of life (DRQOL) in adolescents with T1D from Puerto Rico.	Cross-sectional study 51 Latino Participants (100%) Mean age: 14.78 years (range, 12–17) T1D	Factors associated with suicidal ideation (SI) included depressive symptoms, somatic complaints, and perceived family emotional support, explaining 46% of the variance in SI. Depressive symptoms were the strongest predictor of SI. Factors associated with diabetes-related quality of life (DRQOL) included cognitive alterations, barriers to adherence, family emotional support, and self-efficacy, explaining 61% of the variance in DRQOL. A significant association was found between poorer quality of life and inadequate glycemic control (HbA1c ≥ 7.50), but no direct association was observed between HbA1c as a continuous variable and QOL.	The small sample size limited statistical power, and the study was conducted in Puerto Rico, which may limit generalizability to other Latino populations. The cross-sectional design prevented exploration of causal relationships. Additionally, cultural factors and longitudinal outcomes were not considered.
Hsin et al. (2010) [47] Florida, United States of America To examine the relationship between family involvement, acculturation, and diabetes management (adherence and glycemic control) among Hispanic youth with T1D	Cross-sectional study 111 Latino Participants (100%) Mean age 13.33 years, (SD, 2.82) T1D	Better adherence to diabetes management was associated with less independent youth responsibility for diabetes tasks, more family support for diabetes care, and recent generational status (fewer generations in the US). Better glycemic control was linked to higher parental education and better adherence. Family support mediated the relationship between youth responsibility and adherence, showing that family involvement positively influenced diabetes management in Hispanic youth.	The study lacked sufficient statistical power to test differences across Hispanic/Latino subgroups. While caregiver measures were translated into Spanish, youth measures were not. Acculturation variables, especially linguistic acculturation, may relate differently to health outcomes in areas with less cultural and language diversity. The sample lacked representation of Mexican-American backgrounds, limiting generalizability. Additionally, parents and youths may have different perspectives on diabetes care.
Joiner et al. (2020) [3] Connecticut, United States of America To explore the perceptions and experiences of Latino adolescents with T1D and their parents with limited English proficiency (LEP), focusing on how social and contextual factors, including cultural and linguistic aspects, influence their self-management of T1D.	Qualitative study 24 Latino participants (100%) Mean age: 15.4 years (range, 12–19) T1D	Parents and adolescents initially lacked knowledge and skills for self-management of T1D. Daily tasks, like checking blood glucose and managing insulin, were stressful. T1D interfered with school and social activities. Adolescents desired more independence but felt less autonomous with adult involvement. Family support and spirituality helped in coping, while adolescents found social environments challenging for healthy choices. Traditional family foods were adjusted for T1D, and some parents struggled with balancing culture and diet. Many parents felt isolated, but those with Spanish-speaking healthcare team members felt supported. Parents expressed interest in meeting other Spanish-speaking families for support.	Participants were recruited from a single pediatric subspecialty practice, limiting generalizability. They may not have experienced the full extent of language barriers seen in other settings, which could affect appointment attendance, use of preventive care, and satisfaction. Additionally, the interview guide focused on open-ended narratives, potentially limiting the depth of cultural insights regarding participants’ experiences with T1D.
Knight and Perfect. (2019) [48] No stated specific location from data, United States of America To examine how children and adolescents with T1D glucose levels during and prior to academic assessment contributed to performance on reading, writing, and mathematics tasks.	Randomized Controlled Trial (RCT) 43 Latino participants (51.8%) Mean age: 13.69 years (SD, 2.10) T1D	Hyperglycemia during academic tasks was linked to poorer performance in reading, writing, and math fluency. Youth with glucose levels in the hyperglycemic range performed worse than those in the target range, with levels between 140 mg/dL and 180 mg/dL particularly affecting fluency tasks. Time spent in the hypoglycemic range (below 70 mg/dL) prior to testing was also associated with poorer performance, especially in math and writing. Both short-term glycemic fluctuations and long-term control (HbA1c) significantly impact academic performance in youth with T1D.	The study’s generalizability is limited by its small sample size and recruitment from a single region. Not all participants had complete CGM data. Further research is needed to explore how glucose variability over longer periods affects academic performance and the role of school-based accommodations for students with T1D.
Main et al. (2022) [49] California, United States of America To (1) test associations between parents’ empathic accuracy for their adolescents’ positive and negative emotions and adolescents’ physical and mental health (HbA1c, diabetes self-care, and depressive symptoms) in a predominantly Latinx sample of adolescents with T1D and their parents, and (2) explore how familism values were associated with parent empathic accuracy and adolescent physical and mental health in this population.	Cross-sectional study 68 Latino adolescents (81%) Mean age: 12.74 years (SD, 1.74) T1D	Parents’ empathic accuracy regarding their adolescents’ negative emotions was linked to lower HbA1c, better self-care, and fewer depressive symptoms. Adolescents with parents who understood their negative emotions had better diabetes outcomes. However, empathic accuracy for positive emotions was not significantly related to physical or mental health outcomes. Familism values did not significantly affect parents’ empathic accuracy, but adolescents with higher familism values engaged in better diabetes self-care.	The study was cross-sectional, limiting conclusions about causality. The sample size was modest, and while it included a predominantly Latinx population, findings may not generalize to other groups. Additionally, the study did not explore how other aspects of familism might influence empathic accuracy and diabetes management.
Main et al. (2014) [50] Southwestern, United States of America To examine associations of parent-adolescent relationship quality (parental acceptance and parent-adolescent conflict) with adolescent T1D management (adherence and metabolic control) and depressive symptoms in Latinos and Caucasians.	Cross-sectional study 56 Latino participants (47.5%) Mean age: 12.74 years (SD, 1.64) T1D	Latino adolescents reported lower levels of parental acceptance and higher diabetes-related conflict compared to Caucasians. Despite more conflict, adherence to diabetes management in Latino adolescents was not strongly affected. Better parental acceptance was linked to improved adherence, while family conflict did not significantly reduce adherence. Glycemic control was poor across both groups. Depressive symptoms were more prevalent in Latino adolescents, and higher family conflict correlated with increased depressive symptoms. Familism likely helped Latino adolescents manage diabetes effectively despite conflict.	The study’s cross-sectional design limits causal inferences. The sample was predominantly Mexican-American, which may not generalize to other Latino groups. Self-reported measures may introduce bias, particularly in reporting adherence and family dynamics. Fathers’ perspectives were not included, and acculturation, which could affect parenting behaviors and family dynamics, was not extensively explored.
Mello and Wiebe. (2020) [51] California, United States of America To examine whether disparities in T1D outcomes, specifically in self-management behaviors and HbA1c, exist between Latino and WNH youth, and to assess the role of socioeconomic status (SES) in moderating or explaining these disparities.	Systematic Review The systematic review included a total of 22 studies T1D	Half of the studies showed that Latino youth had poorer glycemic control and self-management, especially in blood glucose monitoring frequency. Socioeconomic status (SES) was a key factor, and when controlled, ethnic differences in T1D outcomes often disappeared. However, in some cases, SES moderated the relationship, with disparities more pronounced at lower SES levels. The review noted inconsistent SES measures across studies, but lower SES was consistently linked to poorer T1D outcomes for Latino youth.	The review noted that findings were inconsistent across studies, particularly regarding the role of SES in explaining or moderating disparities. SES was measured differently across studies, complicating the ability to compare results or identify consistent patterns. Few studies examined socio-cultural factors like acculturation or discrimination, which could contribute to disparities beyond SES.
Mello et al. (2017) [52] Southwest, United States of America To examine ethnic differences in diabetes-related stress and coping among Latino and WNH adolescents with T1D and their mothers; to explore mother-adolescent congruence in perceptions of stress and how these variables were associated with diabetes management outcomes, including adherence and glycemic control.	Cross-sectional study 56 Latinx mother-adolescent dyads (47%) Mean age: 13.24 years (SD, 1.69) T1D	Latino adolescents and their mothers reported fewer issues with low blood glucose compared to WNH families. Latina mothers were less likely to report specific diabetes-related stressors. Dyadic stressor congruence was lower among Latino dyads, indicating that Latina mothers may be less aware of what their adolescents find stressful. Latina mothers viewed their adolescents as less competent in coping with diabetes-related stress, although there were no significant differences in coping strategies. Higher congruence in stress perceptions between mothers and adolescents was linked to better glycemic management (lower HbA1c). Latino dyads with lower congruence and lower maternal appraisals of coping competence had poorer diabetes outcomes, including higher HbA1c.	The sample of Latino dyads was relatively small, limiting the generalizability of the findings to other Latino groups. The study also focused only on mothers, without considering paternal involvement. The results are correlational, meaning that causality cannot be inferred. The study did not measure certain culturally relevant variables directly, such as acculturative stress or Latino cultural values like *familismo*.
Pagán-Torres, Cumba-Avilé and Matos-Melo. (2018) [53] Puerto Rico To examine the psychometric properties of Escala de Autoeficacia para la Depresión en Adolescentes (EADA), a scale measuring emotional self-efficacy for depression in Latino youth with T1D.	Cross-sectional study 51 Latino participants (100%) Mean age: 14.78 years (range, 12–17) T1D	The EADA scale showed excellent internal consistency (α = 0.93) and good concurrent validity, with significant correlations between EADA scores and measures of depression, suicidal ideation, and self-efficacy for diabetes management. Higher self-efficacy was associated with better life satisfaction, greater family support, and better diabetes self-care. The interpersonal dimension of self-efficacy was particularly important for Latino adolescents in managing emotions and seeking support from family and friends.	The sample size did not meet the recommended five cases per item for the total scale, though it did for the subscale level. Despite this, the study followed guidelines to establish reliable alpha estimation with small sample sizes. The non-probabilistic nature of the sample is also a limitation.
Collazo et al. (2013) [54] Connecticut, United States of America To determine whether the establishment of a Spanish Language Diabetes Clinic (SLDC), staffed by Spanish-speaking clinicians, improved metabolic management (HbA1c levels) in Hispanic youth with T1D, compared to similar patients treated in an English Language Diabetes Clinic (ELDC).	Non-randomized controlled trial 21 Latino participants (100%) Mean age: 10.0 years (SD, 0.7) in the SLDC group and 11.0 years (SD, 0.8) in the ELDC group T1D	The SLDC group showed significant improvements in HbA1c (from 8.4% to 7.9%, a decrease of 0.5%, *p* = 0.01), while the ELDC group saw a smaller, non-significant change (from 8.6% to 8.4%, a decrease of 0.2%, *p* = 0.14). At the start, only 23% of SLDC patients had HbA1c levels ≤ 7.5%, compared to 33% in the ELDC. After one year, 48% of SLDC patients achieved HbA1c levels ≤ 7.5%, compared to only 19% in the ELDC (*p* = 0.01).	The small sample size limits the generalizability of the findings. The study was conducted at a single site and for a short duration of one year. Improvements in HbA1c may have been influenced by other factors.
Parker, Lee and Reiboldt. (2013) [55] California, United States of America To investigate the perceptions of youth with diabetes mellitus and their parents/guardians about issues related to eating habits and to identify the relationships between treatment and lifestyle/daily care factors, youth characteristics, and eating habits of youth with DM.	Cross-sectional study 82 Latino participants (65.6%) Mean age: 14.16 years (SD, 2.59) T1D, T2D	Hispanic youth who ate more meals at home showed better eating habits and improved perceptions about healthy eating. Strong parent-child relationships were crucial for promoting healthier eating behaviors, with youth sharing diabetes care with their parents showing better habits than those managing care independently. Hispanic families emphasized the importance of family-centered care, though language barriers sometimes made dietary guidance more challenging.	Small sample size and exclusion of participants who spoke languages other than English or Spanish. The use of non-validated composite scores also weakens the strength of the conclusions.
Pascual et al. (2019) [56] Colorado, United States of America To develop and evaluate a culturally sensitive shared medical appointment (SMA) model for Hispanic pediatric T1D patients.	Experimental, longitudinal study 88 Latino participants (100%) Mean age of the younger group (<12 years): 8.4 years (SD, 2.7); mean age of the older group (≥12 years): 14.6 years (SD, 2) T1D	Younger children (under 12 years) showed significant improvements in HbA1c, decreasing from 9.2% to 8.7% over two years. Their use of insulin pumps increased significantly from 19% at baseline to 60% by year 2. For older participants (12 years and above), HbA1c remained stable from 9.8% at baseline to year 1 but increased to 10.4% by year 2. Insulin pump use increased from 10% to 23% over the same period. The program had a 98% satisfaction rate, with participants preferring culturally sensitive SMA visits over routine care.	The study had a small sample size and was conducted at a single site, limiting the generalizability of the findings. There were challenges in teen participation and attrition, with lower completion rates among older participants. Long-term outcomes and cost-effectiveness were not assessed within the scope of this study.
Piven and Duran. (2014) [57] Texas, United States of America To determine whether an occupation-based intervention could improve diabetes self-management skills in a Mexican-American adolescent with T2D who was exhibiting non-adherent behaviors.	Case study 1 Latino participant (100%) 19 years T2D	The intervention led to improved diabetes self-management. This person adhered to a meal plan, lost 5 pounds, and increased his physical activity from 1 to 7 h per week. He began performing daily glucose checks, achieving an average blood glucose of 145 mg/dL, corresponding to an estimated A1c of 6.6%. The youth became more responsible for his diabetes care, with significant improvements in his self-efficacy (from a score of 1 to 7 on the Diabetes Self-Efficacy Scale) and in his satisfaction with his performance on diabetes-related tasks.	The study involved only one participant, so the findings cannot be generalized. There was no withdrawal phase to assess whether the behavioral changes were maintained over time. The subject initially exhibited denial about his diabetes, which may have influenced the accuracy of early self-reports.
Pyatak et al. (2017) [58] California, United States of America To assess the efficacy of a manualized occupational therapy (OT) intervention, known as REAL Diabetes, designed to improve glycemic management and psychosocial well-being among low-socioeconomic-status (SES), ethnically diverse young adults with T1D or T2D.	Randomized controlled trial (RCT) 63 Latino participants (78%) Mean age: 22.6 years (SD, 3.5) T1D, T2D	Participants in the intervention group (IG) showed significant improvements in HbA1c (a decrease of 0.57%) compared to the control group (CG), which showed an increase of 0.36%. Quality of life (QOL) improved significantly in the IG, with a 0.7-point increase, compared to a 0.15-point improvement in the CG. Habit strength for checking blood glucose increased by 3.9 points in the IG, compared to 1.7 points in the CG. No statistically significant differences were found for other secondary outcomes, although the IG showed trends of improvement in diabetes distress, life satisfaction, and medication adherence.	The study had a relatively small sample size and lacked the statistical power to fully explore secondary outcomes or the effect of intervention dose. There was no long-term follow-up, so it is unclear if the benefits were sustained over time. The study was conducted in a specific urban setting with a high percentage of Latinx participants, which may limit the generalizability of the findings.
Ravi et al. (2021) [59] Colorado, United States of America To assess the uptake rate and clinical impact of CGM on pediatric Medicaid patients with T1D, particularly focusing on glycemic management (HbA1c) after CGM adoption.	Retrospective chart review 264 Latino participants (36.9%) Mean age of participants with some CGM exposure: 10.7 years (SD, 4.5); mean age without CGM exposure: 13.1 years (SD, 4.26) T1D	Hispanic participants were 66.5% less likely to achieve more than 85% CGM usage compared to WNH participants. CGM use was associated with lower A1c levels: Hispanic participants using CGM showed better glycemic management (A1c 8.5%) compared to those who did not use CGM (A1c 9.3%). Although CGM use among Hispanic participants was lower (22.6%), those who did use CGM demonstrated improvements in time in range (TIR) and reduced time in hyperglycemia.	The study had a small number of Hispanic participants using CGM compared to other ethnic groups. Factors such as cultural barriers or prescribing bias may have influenced the lower CGM adoption rate among Hispanic participants.
Raymond et al. (2020) [60] California, United States of America To adapt the CoYoT1 Clinic telehealth group appointment model to a low-SES, publicly insured, racially/ethnically diverse young adult population with T1D.	Randomized controlled trial (RCT) 52 Latino participants (53.1%) Mean age: 19.0 years (SD, 1.7) T1D	Participants in the CoYoT1 telehealth group showed improved clinic attendance, with better adherence to care schedules compared to the control group. Psychosocial outcomes, including reduced diabetes distress and improved self-management behaviors, were also noted. Participants were more satisfied with the flexibility offered by telehealth. However, overall HbA1c levels did not significantly improve over the study period.	No direct limitations are mentioned.
Reitblat et al. (2016) [61] Connecticut, United States of America To describe the experiences of Hispanic adolescents with T1D from both the adolescents’ and their clinicians’ perspectives. Additionally, the study sought to explore how cultural factors, daily management, and clinician-patient interactions influence the life of Hispanic adolescents with T1D.	Qualitative Study 9 Latino participants (100%) Mean age 13.7 years (SD, 1.6) T1D	The study identified key cultural aspects influencing diabetes management, such as traditional Hispanic foods (often carbohydrate-rich) and family closeness, which was both a protective factor and a potential barrier. Adolescents did not see language as a barrier, even when translating for their parents during clinic visits, though clinicians viewed it as a challenge. Adolescents reported that balancing food, insulin, and exercise was a daily struggle and expressed the tension between managing diabetes perfectly and wanting to live without the constraints imposed by diabetes.	The study was limited by its small sample size and potential selection bias, as older adolescents were harder to recruit. Additionally, participants all came from two-parent households, and there was no representation from single-parent homes, which might have offered different perspectives.
Sheikh et al. (2018) [62] Texas, United States of America To assess the rates of diabetes device use (insulin pump and CGM) and their association with glycemic management among youth with T1D in a large, diverse pediatric population.	Cross-sectional study 484 Latino participants (24.3%) Mean age: 13.8 years (SD, 4.2) T1D	Hispanic participants had significantly lower rates of device use (insulin pump and CGM) compared to WNHs. The odds of not using a pump were 1.82 times higher for Hispanic participants compared to WNHs. Similarly, the odds of not using a CGM were 1.62 times higher for Hispanics. CGM use was associated with lower HbA1c for all groups, including Hispanics	The study was limited by its cross-sectional design, which does not establish causality. Additionally, the analysis did not include variables like parental income or education, which are important factors influencing device use
George et al. (2016) [63] No stated specific location from the data; United States of America To assess access to and interest in smartphone technology for managing T1D in primarily Hispanic adolescents and their parents.	Cross-sectional study 37 Latino participants (74%) Mean age: 13.6 years (SD, 2.0) T1D	In total, 98% of adolescents had access to the Internet, and 86% had their own smartphones, while 37% reported using smartphone apps to manage their diabetes, with carbohydrate counting being the most common function (88%). Hispanic adolescents showed moderate to high interest in using technology for T1D management, with girls showing more interest than boys in apps for insulin dose calculation and tracking. Parents demonstrated a higher interest in smartphone apps than adolescents, particularly for glucose tracking, carbohydrate counting, insulin dose calculation, and receiving diabetes-related reminders.	The study only included adolescents attending regular clinic visits, which may not represent those with irregular care access.
Streisand et al. (2002) [64] Puerto Rico To examine self-care behaviors among children and adolescents with T1D living in Puerto Rico, to determine the relationship between self-care and demographic variables, and to investigate the utility of the 24 h recall interview within a Hispanic population.	Cross-sectional study 41 Latino participants and their mothers (100%) Mean age of the children: 12.6 years (SD = 2.9) T1D	Children in Puerto Rico reported relatively good self-care behaviors despite challenging economic conditions. They averaged 5.5 meals per day, with 52% of calories from carbohydrates and 29% from fat, within the recommended ranges. On average, they exercised once per day for 18 min and conducted two blood glucose tests daily. Older children ate less frequently, and girls were more consistent with insulin administration and nutrition habits compared to boys.	The study was limited by its small sample size, which may affect the generalizability of the findings. Limited access to medical records reduced available data on metabolic management for some participants. Additionally, the lack of detailed socioeconomic and psychosocial variables hindered a deeper understanding of self-care behaviors.
Sullivan-Bolya. (2009) [65] Author from Massachusetts, United States of America To improve the cultural and linguistic sensitivity of an established parent-mentor training curriculum for Latino parents of young children newly diagnosed with T1D.	Descriptive, Participatory Action Research Study 4 Latino mothers and 5 children (100%) Age of mothers: range, 38–42 years; age of children range, 7–12 years T1D	The study found that Latino parents face challenges such as language barriers, lack of access to bilingual health providers, and difficulties finding qualified caregivers or school staff for diabetes management. Mothers emphasized the need for culturally sensitive resources, such as bilingual educational materials, and suggested creating diabetes-friendly versions of traditional Latino meals. The adapted intervention, renamed Familias Apoyadas, was well received and considered culturally relevant.	The study had a small sample size of only four mothers, and all were of Puerto Rican origin, which may limit the generalizability of the results. Furthermore, all participants were well educated and acculturated, which may not reflect the broader Latino population.
Taylor et al. (2016) [66] Southeastern, United States of America To assess whether satisfaction with the healthcare provider is related to regimen adherence among primarily minority youth with T1D.	Cross-sectional study 118 Latino participants (70%) Mean age: 13.88 years (SD, 2) T1D	The study found that greater satisfaction with the healthcare provider, particularly in communication and rapport, was associated with better regimen adherence among youth. This association was stronger for girls than boys. Youth satisfaction with the provider was more closely linked to adherence than parent satisfaction, highlighting the importance of the youth-provider relationship. No significant differences in satisfaction or adherence were found based on ethnicity (Hispanic vs. non-Hispanic). The study emphasizes the importance of improving patient-provider relationships to enhance adherence among minority youth.	Results regarding gender were mixed and should be interpreted with caution due to the lack of an interaction effect. Replication with a larger sample is needed, as no causality can be inferred. The study relied on self-reports, and comparing with objective measures of adherence and provider satisfaction could improve understanding. There was no relationship between HbA1c and self-reported adherence. The semi-structured interview format may have led to inflated adherence reports, with social desirability bias possibly affecting gender findings.
Tsai et al. (2022) [67] California, United States of America To assess adolescent and young adult patient attitudes toward, and barriers to, diabetes-specific technology in a predominantly minority, low-socioeconomic-status population, and to investigate whether these differ from those of non-minority youth.	Cross-sectional study 97 Latino participants (56%) Mean age for Latinx English speakers: 17.62 years (SD, 2.78); mean age for Latinx Spanish speakers 14.21 years (SD, 1.91) T1D	Latinx English-speaking participants had more negative attitudes toward both general and diabetes-specific technology compared to Latinx Spanish speakers and non-Latinx English speakers. CGM use was lower in Latinx English speakers (33%) compared to non-Latinx English speakers (61%) and Latinx Spanish speakers (62%). Barriers to technology use were similar across groups, but Latinx English speakers expressed more nervousness about using diabetes devices. Participants with higher HbA1c levels tended to have more negative attitudes toward technology, with Latinx English speakers having the highest HbA1c (9.69%).	The study’s cross-sectional design limits the ability to establish causality. The study was conducted in a single hospital setting, and the findings may not generalize to other regions. The sample size for certain subgroups (e.g., Latinx Spanish speakers) was relatively small. The questionnaires used to assess attitudes and barriers were adapted from adult surveys, and may not fully capture pediatric experiences.
Tucker et al. (2018) [68] Southwestern, United States of America To examine the associations between adolescents’ disclosure of and secrecy about their T1D management with parental knowledge and the impact of these on diabetes outcomes (such as adherence, glycemic management, and depressive symptoms) in both WNH and Latino youth.	Cross-Sectional Study 56 Latino participants (47.5%) Mean age 13.24 years (SD, 1.69) T1D	Adolescent disclosure (sharing information with parents) was strongly associated with better glycemic management (lower HbA1c), better adherence, and fewer depressive symptoms for both Latino and WNH youth. Secrecy (withholding information from parents) was linked to poorer adherence and higher depressive symptoms, particularly impacting glycemic management in those who kept secrets from their fathers. No significant ethnic differences were found regarding the impact of disclosure and secrecy, although Latino adolescents disclosed less to their fathers compared to WNH adolescents.	The study’s cross-sectional design limited the ability to assess causality. Most data were collected from adolescents, which may have introduced bias in reports of disclosure and secrecy. Fathers did not participate in the study, and all information about fathers was based on adolescent reports.
Vajravelu et al. (2021) [69] No stated study location; authors from the United States of America To investigate patterns and predictors of treatment escalation within 5 years of metformin monotherapy initiation for youth with T2D in clinical practice.	Cohort retrospective study 159 Latino participants (19%) Mean age: 15 years (range, 10–18) T2D	Hispanic youth were nearly twice as likely to experience treatment escalation compared to White youth (HR 1.83, 95% CI 1.28–2.61). This escalation included both insulin and non-insulin antihyperglycemics. Older age at diagnosis and adherence to metformin were also strongly associated with treatment escalation in the Hispanic population. Hispanic participants, like Black participants, were more likely than White participants to escalate to insulin rather than non-insulin antihyperglycemics.	The study’s limitations included reliance on medical claims data, which may have missed certain clinical details. The cohort was also limited to commercially insured patients, so the findings may not be generalizable to populations with government insurance or uninsured groups. Additionally, HbA1c data were not available for the entire cohort, limiting insights into glycemic management across different ethnicities.
Delamater et al. (1999) [70] No stated study location; authors from the United States of America To quantify the degree of risk for poor metabolic management among Black, Hispanic, and White children and adolescents with diabetes	Retrospective chart review 145 Latino participants (56.4%) Mean age: 12.5 years (SD, 5) T1D	Hispanic youth had worse glycemic management than WNH youth but better management than Black youth. Hospitalizations for DKA were similar between Hispanic (15.2%) and White (18.2%) youth and lower than in Black youth (23.5%), with no significant difference in DKA risk. Secondary analysis showed higher prior HbA1c in Hispanic youth compared to White youth. Despite low socioeconomic status, access to private insurance among Hispanic youth was higher than among Black youth, possibly contributing to their relatively better metabolic outcomes.	Study limitations included broad ethnic categorization based on medical records, potential misclassification of diabetes type, and use of multiple laboratories requiring HbA1c standardization. Differences between clinical sites and lack of data on clinic visit frequency may have introduced variability. The cross-sectional design also limited the assessment of causal relationships.
Lipman et al. (2020) [71] Pennsylvania, United States of America To quantify racial and ethnic disparities in a large urban pediatric center, by comparing treatment modalities, clinical outcomes and appointment attendance in Black non-Hispanic BNH, vs. WNH and Hispanic children with T1D while examining the contribution of insurance status (as a proxy for SES) to these disparities.	Retrospective chart review 107 Latino participants (8%) Mean age: 14 years (range, 11.6–15.6) T1D	Hispanic youth had higher HbA1c levels than WNH youth but lower than BNH youth. CGM use was lower among Hispanic youth (53%) compared to WNH youth (68%) but higher than BNH youth (39%). Disparities in CGM use persisted after adjusting for socioeconomic status. Insulin pump use among Hispanic youth was 47%, this being significantly lower than WNH youth (72%), but higher than that among BNH youth (34%). Hispanic youth experienced more hospitalizations and emergency department visits than WNH youth but fewer hospitalization and visits than BNH youth (18% and 15%). Higher missed appointment rates and lower technology use may have contributed to increased healthcare utilization.	The study’s limitations included its retrospective design, limiting assessment of causal relationships and preventing evaluation of whether diabetes technologies were offered or declined. Socioeconomic status was approximated using insurance type, which may not fully capture socioeconomic factors. As a single-center study, findings may not be generalizable to other settings. The lack of detailed data on family acceptance of treatment options and potential unmeasured social determinants of health may have introduced bias.
Lado and Lipman. (2016) [72] Authors from United States of America. To review the racial and ethnic disparities in the incidence, treatment, and outcomes of children with T1D.	Literature review T1D	Hispanic youth reported fewer daily blood glucose checks (5.6) than WNH (6.1) but more checks than BNH (5.4). They had lower insulin pump use (12.3%) than WNH (26.3%), with disparities persisting after SES adjustment. CGM use was also lower than that for WNH. HbA1c was higher in Hispanic youth (8.7%) than WNH (8.4%) but lower than in BNH (9.6%). DKA (12%) and severe hypoglycemia (6%) rates were higher than for WNH but lower than for BNH.	No direct limitations are mentioned.

## Data Availability

Data are available upon request.

## Abbreviations

The following abbreviations are used in this manuscript:

PubMed	US National Library of Medicine
CINAHL	Cumulative Index to Nursing and Allied Health Literature
LILACS	Latin American and Caribbean Health Sciences Literature
ERIC	Education Resources Information Center
JBI	Joanna Briggs Institute
ADCES7	Association of Diabetes Care and Education Specialists framework
CGM	Continuous glucose monitoring
HbA1c	Glycated hemoglobin
DSME	Diabetes self-management education
PRISMA-ScR	Preferred Reporting Items for Systematic Reviews and Meta-Analyses Extension for Scoping Reviews
PCC	Population, concept and context
DeCS-MeSH	Descriptors in Health Sciences—Medical Subject Headings
T1D	Type 1 diabetes
T2D	Type 2 diabetes
SES	Socioeconomic status
VPG	Virtual peer group
MPR	Medication possession ratio
SLDC	Spanish Language Diabetes Clinic
SMA	Shared medical appointment
CoYoT1	Colorado Young Adults with T1D
DKA	Diabetes ketoacidosis
YA	Young adults
NH	Non-Hispanic
SCI	Self-Care Inventory
CES-D	Center for Epidemiologic Studies Depression Scale
DRS	Diabetes-related stigma
SS	Social stigma
IS	Internalized stigma
WNH	White Non-Hispanic
SI	Suicidal ideation
DRQOL	Diabetes-related quality of life
LEP	Limited English Proficiency
EADA	Escala de Autoeficacia para la Depresión en Adolescentes
ELDC	English Language Diabetes Clinic
DM	Diabetes mellitus
QOL	Quality of life
BNH	Black Non-Hispanic

## Appendix A

### Search Strategies

PUBMED: (“Hispanic or Latino”[Mesh] OR Hispanic[tw] OR Latino[tw] OR Latinx[tw] OR Latina[tw]) AND (“Diabetes Mellitus”[Mesh] OR “Diabetes Mellitus, Type 1”[Mesh] OR “Diabetes Mellitus, Type 2”[Mesh] OR diabet*[tw] OR “type 1 diabetes”[tw] OR “type 2 diabetes”[tw]) AND (“Self Care”[Mesh] OR “Self-Management”[Mesh] OR “Culturally Competent Care”[Mesh] OR “Communication Barriers”[Mesh] OR “Shared Medical Appointments”[Mesh] OR “Patient Compliance”[Mesh] OR “Treatment Adherence and Compliance”[Mesh] OR “self care”[tw] OR “self-management”[tw]) AND (Adolescent[Mesh] OR adolescen*[tw] OR “Young Adult”[Mesh] OR Child[Mesh] OR child[tw] OR “Child, Preschool”[Mesh] OR preschool[tw] OR teenager*[tw] OR Youth[tw] OR Infant[Mesh] OR infan*[tw] OR Pediatrics[Mesh] OR pediatric*[tw])
SCOPUS: (Hispanic OR Latino OR Latinx OR Latina) AND (“Diabetes Mellitus” OR “Diabetes Mellitus Type 1” OR “Diabetes Mellitus Type 2” OR diabet* OR “type 1 diabetes” OR “type 2 diabetes”) AND (“Self Care” OR “Self-Management” OR “Culturally Competent Care” OR “Communication Barriers” OR “Shared Medical Appointments” OR “Patient Compliance” OR “Treatment Adherence and Compliance”) AND (Adolescent OR adolescen* OR “Young Adult” OR Child OR “Child, Preschool” OR teenager* OR Youth OR Infant OR Pediatrics)
The Cochrane Library: (Hispanic OR Latino OR Latinx OR Latina) AND (“Diabetes Mellitus” OR “Diabetes Mellitus Type 1” OR “Diabetes Mellitus Type 2” OR diabetes OR “type 1 diabetes” OR “type 2 diabetes”) AND (“Self Care” OR “Self-Management” OR “Culturally Competent Care” OR “Communication Barriers” OR “Shared Medical Appointments” OR “Patient Compliance” OR “Treatment Adherence and Compliance” OR self care OR self-management) AND (Adolescent OR adolescen OR “Young Adult” OR Child OR “Child, Preschool” OR preschool OR teenager OR Youth OR Infant OR Pediatrics OR pediatric)
LILACS: (Hispanic OR Latino OR Latinx OR Latina) AND (“Diabetes Mellitus” OR “Diabetes Mellitus Type 1” OR “Diabetes Mellitus Type 2” OR diabetes OR “type 1 diabetes” OR “type 2 diabetes”) AND (“Self Care” OR “Self-Management” OR “Culturally Competent Care” OR “Communication Barriers” OR “Shared Medical Appointments” OR “Patient Compliance” OR “Treatment Adherence and Compliance” OR self care OR self-management) AND (Adolescent OR adolescen OR “Young Adult” OR Child OR “Child, Preschool” OR preschool OR teenager OR Youth OR Infant OR Pediatrics OR pediatric)
CINAHL: (Hispanic or Latino OR Hispanic OR Latino OR Latinx OR Latina) AND (Diabetes Mellitus OR Diabetes Mellitus Type 1 OR Diabetes Mellitus Type 2 OR diabet* OR type 1 diabetes OR type 2 diabetes) AND (Self Care OR Self-Management OR Culturally Competent Care OR Communication Barriers OR Shared Medical Appointments OR Patient Compliance OR Treatment Adherence and Compliance OR self care OR self-management) AND (Adolescent OR adolescen* OR Young Adult OR Child OR child OR Child, Preschool OR preschool OR teenager* OR Youth OR Infant OR Pediatrics OR pediatric*)
Web of Science: (Hispanic OR Latino OR Latinx OR Latina) AND (“Diabetes Mellitus” OR “Diabetes Mellitus Type 1” OR “Diabetes Mellitus Type 2” OR diabet* OR “type 1 diabetes” OR “type 2 diabetes”) AND (“Self Care” OR “Self-Management” OR “Culturally Competent Care” OR “Communication Barriers” OR “Shared Medical Appointments” OR “Patient Compliance” OR “Treatment Adherence and Compliance”) AND (Adolescent OR adolescen* OR “Young Adult” OR Child OR “Child, Preschool” OR teenager* OR Youth OR Infant OR Pediatrics)
ERIC: (Hispanic OR Latino OR Latinx OR Latina) AND (“Diabetes Mellitus” OR “Diabetes Mellitus Type 1” OR “Diabetes Mellitus Type 2” OR diabetes OR “type 1 diabetes” OR “type 2 diabetes”) AND (“Self Care” OR “Self-Management” OR “Culturally Competent Care” OR “Communication Barriers” OR “Shared Medical Appointments” OR “Patient Compliance” OR “Treatment Adherence and Compliance” OR self care OR self-management) AND (Adolescent OR adolescen OR “Young Adult” OR Child OR “Child, Preschool” OR preschool OR teenager OR Youth OR Infant OR Pediatrics OR pediatric)
